# Subcellular Roles of Glutathione in Mediating Plant Defense during Biotic Stress

**DOI:** 10.3390/plants9091067

**Published:** 2020-08-20

**Authors:** Bernd Zechmann

**Affiliations:** Center for Microscopy and Imaging, Baylor University, One Bear Place #97046, Waco, TX 76798, USA; Bernd_Zechmann@baylor.edu

**Keywords:** antioxidants, arabidopsis, chloroplasts, glutathione, mitochondria, reactive oxygen species, resistance

## Abstract

Glutathione and reactive oxygen species (ROS) play important roles, within different cell compartments, in activating plant defense and the development of resistance. In mitochondria, the accumulation of ROS and the change of glutathione towards its oxidized state leads to mitochondrial dysfunction, activates cell death, and triggers resistance. The accumulation of glutathione in chloroplasts and peroxisomes at the early stages of plant pathogen interactions is related to increased tolerance and resistance. The collapse of the antioxidative system in these two cell compartments at the later stages leads to cell death through retrograde signaling. The cytosol can be considered to be the switchboard during biotic stress where glutathione is synthesized, equally distributed to, and collected from different cell compartments. Changes in the redox state of glutathione and the accumulation of ROS in the cytosol during biotic stress can initiate the activation of defense genes in nuclei through pathways that involve salicylic acid, jasmonic acid, auxins, and abscisic acid. This review dissects the roles of glutathione in individual organelles during compatible and incompatible bacterial, fungal, and viral diseases in plants and explores the subcelluar roles of ROS, glutathione, ascorbate, and related enzymes in the development of resistance.

## 1. Introduction

**Figure 1:**
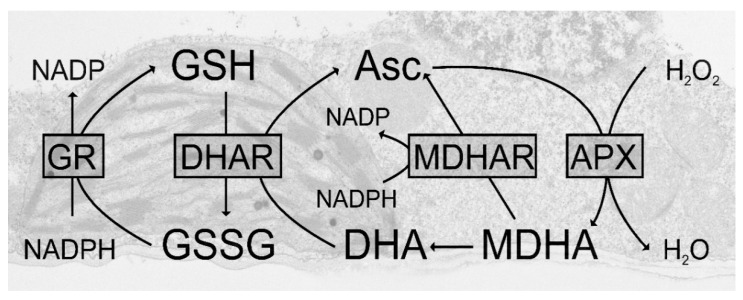
Ascorbate-glutathione cycle. Within the plant cell hydrogen peroxide (H_2_O_2_) is detoxified by ascorbate peroxidase (APX). Through this reaction, the reduced form of ascorbate (Asc) is oxidized to monodehydroascorbate (MDHA). MDHA is then further reduced by monodehydroascorbate reductase (MDHAR) to Asc, or reacts to dehydroascorbate (DHA). DHA is reduced by dehydroascorbate reductase (DHAR) to Asc. Through this reaction, the reduced form of glutathione (GSH) is oxidized to glutathionedisulfide (GSSG). GSSG is then reduced by glutathione reductase (GR) to GSH. The electron acceptor NADP is regenerated during the reduction of MDHA and GSSG by the respective enzymes.

Plants are constantly attacked by a wide range of pathogens that threaten their existence. Thus, they have developed a sophisticated network of defense to protect themselves from developing diseases [1,2,3]. Physical adaptations, such as thickening of cell walls, keep the invaders outside [4]. For some pathogens these barriers are ineffective, and once inside, the plant activates chemical pathways to fight the dangerous invaders [1,2,3,4,5,6,7,8]. Some pathogens, such as necrotrophic fungi and bacteria, kill their hosts and prey on the nutrients of the plants [9,10,11]. Others, such as biotrophic fungi or bacteria live in a mutualistic relationship with the plant from which both partners benefit without killing each other [12,13,14,15]. Some fungi switch from a biotrophic to a necrotrophic relationship during their lifetime [16,17]. Bacteria have a similar relationship with the plant and can be necrotrophic, biotrophic, or both. They mostly live in the apoplastic space where they prey on the nutrients of the plant. However, some can also enter its cells [14,18,19]. Viruses, on the other hand, which cannot live on their own, depend on the machinery of living cells and generally keep their hosts alive as long as possible. Thus, viral infections are often latent in plants and rarely end in the death of the plant [20,21]. Viruses enter plants through wounds or cracks or get injected by aphids. The virus then spreads rapidly throughout the whole plant, multiplies inside its cells, and alters their metabolism which leads to impairments in growth and reproduction or death [20,22,23,24,25]. Considering the wide range, amount, and interactions of pathogens with plants it becomes obvious that plant pathogen interactions involve many complex pathways on the cellular level. Over the past few decades, many (groups of) agents have established themselves as important strategic players in the defense of plants against pathogens such as antioxidants, lipids, reactive oxygen species (ROS), salicylic acid (SA), jasmonic acid (JA) and many more [8,26,27,28,29]. Among these agents glutathione plays a very important dual role. It keeps ROS, which often accumulate during biotic stress, under control and therefore, reduces damage to the cells [27,30]. It also activates defense pathways against pathogens by mediating between ROS, SA, JA, and ethylene [27,30,31,32,33,34,35,36,37,38]. Pathogen infections induce a wide variety of ROS, such as singlet oxygen, superoxide anions, hydrogen peroxide, hydroxyl, and nitrous oxides, which can accumulate in all cell compartments. If not detoxified ROS can oxidize lipids, inhibit enzymes, inactivate biomolecules, and damage proteins, RNA, and DNA. ROS are detoxified either through enzymes (e.g., catalase, peroxidases, superoxide dismutase, etc.) or through non-enzymatic antioxidants such as glutathione and ascorbate. Glutathione and ascorbate can detoxify ROS, either directly or indirectly, through the ascorbate-glutathione cycle [39,40,41,42] (Figure 1).

**Figure 2:**
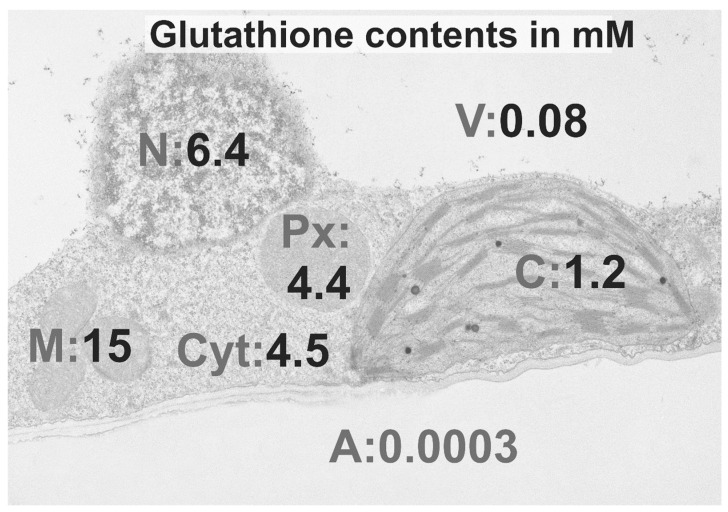
Compartment-specific concentrations of glutathione in non-stressed Arabidopsis Col-0 plants. Transmission electron micrograph of a leaf cell with overlay of compartment-specific concentrations of glutathione for chloroplasts (C), the cytosol (Cyt), mitochondria (M), nuclei (N), peroxisomes (Px), and the vacuoles (V). Glutathione contents were determined by using a combination of biochemical measurements and cytohistochemical detection of glutathione by transmission electron microscopy [60]. As glutathione could not be detected in the apoplast (A) with cytohistochemical methods, concentrations in the apoplast were taken from a study where glutathione concentrations were measured with biochemical methods after extraction of soluble apoplastic glutathione [45].

Inside non-stressed cells glutathione occurs up to 90% in its reduced form (GSH) and up to 10% in its oxidized form (GSSG). Glutathione contents vary strongly within cells (Figure 2) with highest contents in mitochondria (15 mM) followed by nuclei (6.4 mM), the cytosol (4.5 mM), peroxisomes (4.4 mM), chloroplasts (1.2 mM), vacuoles (0.08 mM), and the apoplast (0.0003 mM). Stress situations, such as the infection of plants with pathogens, often induce oxidative stress which changes glutathione contents and the ratio of glutathione toward the oxidized form [33,43,44]. Such changes in the redox state of the cell can then signal stress and activate plant defense through different defense pathways [33]. During compatible plant pathogen interaction, when the invader spreads systemically throughout the whole plant, the main role of glutathione seems to be to protect the plant against oxidative damage by keeping ROS under control, which could otherwise lead to the death of the plant [27]. Nevertheless, glutathione, ascorbate, and antioxidative enzymes often fail to efficiently detoxify ROS during susceptible plant pathogen interactions [43,44,45,46], which leads to the spread of the pathogen throughout the plant and to the development of systemic symptoms [47,48]. During incompatible plant pathogen interaction, when the pathogen is unable to spread throughout the plant, the main role of glutathione seems to be stress signaling and activation of plant stress responses [27,30,32,33,34,35]. Many incompatible plant pathogen interactions are characterized by a hypersensitive response (HR), which includes the accumulation of ROS and subsequently local cell death, which limits the spread of the diseases and activates defense pathways that lead to resistance [27,30,49,50]. Glutathione contents often increase during HR and the redox state becomes more oxidized, which switches on plant defense genes and results in resistance [33,44]. These two roles of glutathione (protection against ROS and mediation of the activation of defense genes) are essential for the protection of plants during compatible and incompatible reactions. For example, during compatible infection of tomato plants with *Pseudomonas syringae*, decreased GSH contents and the accumulation of GSSG were observed in the susceptible species, while GSH pool homeostasis was maintained in the resistant cultivar [43]. During the mutualistic fungal infection of *Blumeria graminis* glutathione contents remained largely unaffected [51] or were too low to keep the accumulation of H_2_O_2_ under control [45,46]. GSH contents on the other hand strongly accumulated in Arabidopsis plants during the infection with the necrotrophic fungi *Botrytis cinerea* in order to counteract oxidative stress induced to kill the plant [52]. During Zucchini Yellow Mosaic Virus (ZYMV)-infection, a tolerant pumpkin species showed a much stronger accumulation of glutathione 14 days post inoculation (dpi) and a lack of symptoms development [53] when compared to the susceptible species, which showed much lower glutathione accumulation and developed stronger symptoms [54,55]. The importance of glutathione in the development of resistance against pathogen infections is further supported by several studies that demonstrated that the artificial elevation of glutathione resulted in the activation of defense genes, decreased virus contents, and reduced symptom development [32,33,34,35,55,56,57,58,59].

Most knowledge of the above-mentioned pathways has been obtained from investigations of whole organs or whole plants. Nevertheless, glutathione metabolism is highly compartment specific. It is synthesized in chloroplasts and the cytosol [61], but it is degraded in the apoplast, the cytosol, and in vacuoles [62,63,64]. After production, glutathione transporters distribute glutathione among all cell compartments [65]. In non-stressed cells glutathione contents differ strongly among different cell compartments with highest concentrations in mitochondria, followed by nuclei, the cytosol, peroxisomes, chloroplasts, vacuoles, and the apoplast [66]. This distribution changes drastically during stress situations, and depending on the stressor different compartments can be affected differently. For example, stress that targets chloroplasts (e.g., high light, drought) causes massive changes in glutathione contents in these cell compartments while other compartments are not or less affected [66,67,68]. Thus, subcellular changes in glutathione contents can give valuable information about the response of plants to the respective stress and can be used as a subcellular stress marker. The aim of this review is to summarize the data available in the literature about the subcellular role of glutathione during biotic stress situations and to dissect the mechanisms it plays in the protection of plants against different pathogens.

## 2. Fungal Infections

Fungi invade plant tissue by spreading hyphae from the surface throughout the infected tissue. Openings like stomata and cracks are preferred points of entry but the hyphae can also grow into intact epidermis cells [9,10,11,12,69]. Plant-fungi-interactions can be generally divided into two major groups: biotrophic and necrotrophic. Biotrophic interactions are characterized by a mutualistic relationship between the plant and the fungi. The fungi feeds from nutrients that the plant provides. In some cases, the fungi also provides benefits to the plant [12,13,15]. The underlining mechanisms of such interactions are that the fungi masks its appearance and that the plant does not recognize its presence. Thus, the plant does not activate its defense or reduces its defenses to an extent that it keeps the fungal infection under control. Some biotrophic fungi can also turn into necrotrophic fungi during their life span [13,17,69,70]. Necrotrophic interactions are characterized by the complete destruction of the plant by the fungi. The fungi then preys on the dead plant material [9,10,11]. The rapid accumulation of ROS, also called HR, in the early stages of plant-fungi interaction is a common response of plants to fight off fungal pathogens [49,50]. As GSH detoxifies ROS, it is not surprising that changes in glutathione contents are commonly observed during these stages of fungal infections [44,45,46,52]. Nor is it surprising that the artificial elevation of glutathione through external application of GSH and L-2-oxothiazolidine-4-carboxylic acid (OTC) enhanced basal resistance against powdery mildew infection [34].

On the cellular level, the first line of defense in plants against fungi is the apoplast. A strong accumulation of ROS in the apoplast is a common response of the plant against the fungi [52], which is aimed to directly kill the hyphae and to activate plant defense [4,71]. During these early stages of infection, glutathione plays important signaling roles in the apoplast. In oat and barley plants, a strong increase of glutathione and ascorbate contents in the apoplast was associated with resistance against the biotrophic fungi powdery mildew (*Blumeria graminis*). Decreased glutathione and ascorbate levels but higher activity of superoxide dismutase, catalase, APX, GR, DHAR, MDHAR, and GR were found in the apoplast of the susceptible plants [45,46]. These results indicate that glutathione (and ascorbate) fulfill important signaling roles in the apoplast leading to resistance. Similar results were obtained in tobacco plants inoculated with powdery mildew where the artificial elevation of glutathione through injection of GSH into the apoplast enhanced basal resistance against *Euoidium longipes* [34]. The essential role of glutathione in the apoplast for signaling plant defense against fungal pathogens is supported by the absence of glutathione in the apoplast during necrotrophic fungal infection, which results in plant death [52]. Even though a strong accumulation of H_2_O_2_ was observed in the apoplast at the early stages of infection of Arabidopsis plants with the necrotrophic fungi *Botrytis cinerea*, glutathione remained absent from the apoplast throughout the infection [52]. Interestingly, glutathione contents in the apoplast were not detected, or were very low, during *Blumeria graminis* infection and could not control H_2_O_2_ accumulation in the HR type of resistance [45,46]. Thus, it seems that, in the apoplast, glutathione and/or its redox state (which becomes more oxidized during HR) serves important roles as a defense-signaling agent rather than as an antioxidant antagonizing ROS accumulation during fungal infection.

As already pointed out, glutathione does not play important signaling and protective roles in the apoplast during necrotrophic *Botrytis cinerea* infection in Arabidopsis [52]. In this study the authors put a drop of fungal spores on an Arabidopsis leaf and monitored glutathione levels in cells at, and adjacent to, the infection site. While the infection site showed strong symptoms such as necrosis 48 h post inoculation (hpi), the area adjacent to the infection site showed only minor symptoms such as chlorosis at this stage. All cell compartments, except mitochondria, showed a strong increase in glutathione contents, 12 hpi and 48 hpi at the infection site and 48 hpi in cells adjacent of the infection site (Table 1). In contrast, ascorbate contents were strongly decreased at the infection site in all cell compartments [52]. The decrease of glutathione and ascorbate contents in mitochondria, at the later stages of infection, was correlated with an accumulation of H_2_O_2_ [52]. A similar situation was also found in *Botrytis cinerea* infected tomato plants. In this case, a strong decrease of glutathione and ascorbate contents in mitochondria was accompanied with the accumulation of GSSG, DHA, and pathogen induced senescence [44]. On top of that, the activity of APX, DHAR, and MDHAR was strongly reduced in mitochondria as well. Based on these studies, it can be concluded that the breakdown of the antioxidative system in mitochondria leads to the accumulation of ROS during *Botrytis cinerea* infection. Such circumstances then lead to the loss of outer membrane integrity of mitochondria and the release of proteins to the cytosol, which results in programmed cell death [72,73,74].

Another interesting aspect during *Botrytis cinerea* infection was that chloroplasts showed the strongest increase in glutathione contents throughout the infection, while ascorbate contents depleted in chloroplasts. Despite the accumulation of glutathione in chloroplasts, H_2_O_2_ accumulated at the later stages in this cell compartment indicating that GSH was not able to keep H_2_O_2_ under control [52]. Similar results were collected in tomato plants infected with the fungal pathogen *Botrytis cinerea*. In this case, the collapse of the antioxidative system (decreased glutathione and ascorbate contents, decreased MDHAR activity) and the accumulation of GSSG and DHA in chloroplasts and peroxisomes was associated with pathogen induced leaf senescence [44]. The results of these studies clearly demonstrate that high levels of glutathione in chloroplasts and peroxisomes counteract ROS accumulation during *Botrytis cinerea* infection. When glutathione fails to keep ROS in chloroplasts and peroxisomes under control systemic symptoms and cell death are initiated through retrograde signaling [44,52,75,76]. Besides the accumulation of glutathione in chloroplasts during pathogen attack, its redox state is also of great importance for the development of resistance. The accumulation of GSSG and DHA in mitochondria, chloroplasts, and peroxisomes was correlated with symptom development and senescence in *Botrytis cinerea* infected tomato plants [44]. It has been demonstrated that the overexpression of plastidial GR increased total glutathione contents and enhanced resistance in wheat against *Blumeria graminis* [51]. It was concluded by the authors that the overexpression of GR increased the ratio of glutathione towards GSH. The change of the cytosolic redox state reduced NPR1 to a monomer, which subsequently led to the activation of SA mediated *PR* genes when imported into the nucleus [51].

Summing up, apoplastic glutathione contents are involved in signaling and activating plant defense during biotrophic, but not necrotrophic, plant fungi interaction. The breakdown of the antioxidative system in mitochondria during necrotrophic plant fungal interactions results in the accumulation of ROS, disruption of the integrity of mitochondria, and the release of proteins into the cytosol, which triggers cell death. The most important roles of glutathione in chloroplasts and peroxisomes during fungal infection is to keep ROS under control and to activate plant defense. When glutathione fails to keep ROS in chloroplasts and peroxisomes under control, systemic symptoms and cell death are initiated through retrograde signaling. When glutathione accumulates and its redox state is maintained in the chloroplasts, plant defense is activated through SA mediated pathways.

## 3. Bacterial Infections

Bacteria are single-celled organisms that enter plants through wounds or cracks and live off the nutrients provided by the plant. In mutualistic relationships, bacteria can supply nutrients to the plant and even offer first line defense against other pathogens [14,15,18]. Necrotrophic bacteria, on the other hand, feed off the nutrients from the plant while the toxins they release slowly kill the plant [9,19]. Most bacteria live in the apoplastic space where they live off the nutrients of the plant. However, some can also enter into cells [14,18,19,77].

Considering that most bacteria live in the apoplast of the plant, one would expect that changes in apoplastic glutathione contents would be a logical response of plants during bacterial infections. Nevertheless, despite a massive increase of ROS throughout the leaf, glutathione and ascorbate could not be detected in the apoplast of Arabidopsis plants inoculated with *Pseudomonas syringae* [75]. These results indicate that glutathione in the apoplast does not play an important role to keep H_2_O_2_ under control during bacterial infection. Inside the cell, strong changes in glutathione contents could be observed in Arabidopsis plants after a solution of *Pseudomonas syringae* was injected into the leaves (Table 2) [75]. While symptoms, such as yellowing and ROS accumulation in plants inoculated with the avirulent strain, were observed as early as 12 hpi, plants inoculated with the virulent strain showed first symptoms at a later stage (24 hpi). This was also accompanied by a much lower accumulation of ROS and slower development of symptoms throughout the experiment. Accordingly, plants infected with the avirulent strain showed a strong increase of glutathione and ascorbate, 12 hpi in all cell compartments, while the virulent strain showed delayed glutathione accumulation, 24 hpi (Table 2). The infection of tomato plants with *Pseudomonas syringae* induced a strong drop of GSH contents and the accumulation of GSSG which was accompanied with the development of necrotic lesions. Such effects could not be found in the resistant cultivar, which did not develop lesions and where glutathione and ascorbate homeostasis was maintained. Ascorbate contents and the activity of GR, APX, and glutathione peroxidase in the resistant cultivar mostly mimicked the situation in the controls [43]. Thus, these results demonstrate that elevated glutathione contents and the accumulation of ROS play an important role in activating plant defense against avirulent *Pseudomonas syringae* infection. Avirulence is generally characterized by an oxidative burst starting very early during the infection. This HR leads to local cell death which inhibits the spread of the disease and also activates plant defense throughout the plant [27,30,49,50]. In this respect, it is interesting that glutathione contents strongly increased in all cell compartments 12 hpi, which correlated well with the occurrence of ROS and first symptoms. At 24 hpi, when first necrotic lesions and a strong accumulation of ROS appeared on the leave infected with the avirulent strain, glutathione contents in mitochondria and chloroplasts dropped relatively to the situation at 12 hpi, while it increased in all other cell compartments [75]. As demonstrated in *Pseudomonas syringae* infected Arabidopsis mutants, an appropriate oxidized glutathione status and the depletion of glutathione contents triggered by oxidative stress is responsible for the induction of resistance through the accumulation of SA and the induction of *PR* genes [33]. Thus, the above described results clearly demonstrate that the ROS induced depletion of glutathione in mitochondria and chloroplasts and its change towards a more oxidative state is the main trigger for plant defense against avirulent *Pseudomonas syringae* infection [33,73]. In this respect it has also been demonstrated that mitochondrial dysfunction induced by ROS, activates cell death, initiates plant defense, and triggers resistance [72,74].

It is also important to mention that glutathione contents showed a massive increase in peroxisomes in the early stages of virulent and avirulent *Pseudomonas syringae* infection (Table 2). The importance of peroxisomes for plant defense has also been highlighted in a study using catalase deficient Arabidopsis mutants infected with a virulent strain of *Pseudomonas syringae* [33]. These mutants contain enhanced photorespiratory H_2_O_2_ levels which trigger the induction of pathogen related defense genes and cell death [21,33,73,78,79]. The activation of plant defense during *Pseudomonas syringae* infection under these circumstances was correlated with the accumulation of GSSG and the activation of SA signaling, which led to reduced bacterial growth [33]. Blocking glutathione oxidation and accumulation, strongly reduced plant defense, inhibited lesion formation, and led to bacterial growth similar to the control [33]. These results clearly indicate that in situations of catalase deficiency in peroxisomes, GSSG and H_2_O_2_ accumulate and activate plant defense through SA-mediated pathways.

Summing up, glutathione does not play important roles in the apoplast to activate or signal plant defense against bacterial infections. The depletion of glutathione in mitochondria and chloroplasts and its change towards a more oxidative state is the main trigger for plant defense against avirulent *Pseudomonas syringae* infection. The accumulation of glutathione in peroxisomes and a shift towards its oxidized state activates plant defense through SA-mediated pathways.

## 4. Viral Infections

Unlike bacteria and fungi, viruses cannot survive, reproduce, or spread without a host. They do not prey on nutrients from the plant but use its cells for reproduction. Thus, viruses tend to not kill their hosts but impair their metabolism which can lead to stunted growth, discoloration of leaves, deformations of fruits, etc. [22,56,80,81,82]. Many studies have shown that elevated glutathione levels lead to improved disease resistance against viruses. The artificial increase of glutathione by OTC and sulfur treatment inhibited symptom development and decreased virus contents in Zucchini Yellow Mosaic Virus (ZYMV)-infected pumpkin plants [54,55] and Tobacco Mosaic Virus (TMV) infected tobacco plants [58,59], respectively. OTC pretreatment also induced partial protection against Plum Pox virus (PPV) in pea plants [56] and against TMV in tobacco plants [35]. Elevated glutathione contents could also be correlated in tobacco plants with an increase of defense-related genes and proteins [35,58,59]. Additionally, a tolerant pumpkin species, which did not develop symptoms but contained virions in the sap and typical ultrastructural alterations of ZYMV-disease, showed a much stronger accumulation of glutathione in leaves [53] than the susceptible species, which showed strong symptoms of ZYMV 14 dpi [54]. The mechanisms behind the suppressing effects of glutathione on symptom development and virus contents lay in the activation and modulation of defense genes. The application of glutathione in Arabidopsis plants led to the accumulation of the resistance marker protein PR-1 while the inhibition of glutathione synthesis suppressed the expression of the *PR-1* gene [83]. Treating tobacco plants with sulfur increased glutathione contents, activated the expression of several *PR* genes, and decreased symptom development and virus contents [58,59]. Similar results were obtained in tobacco plants infected with TMV where the artificial elevation of glutathione through external application of GSH and OTC induced resistance through the overexpression of several *PR* genes and elevated levels of SA [35]. A similar study in tobacco plants demonstrated that high glutathione levels could be correlated with the overexpression of several defense related genes and proteins. It also activated plant defense through *PR-1* gene induction and SA accumulation [32,33,84]. These results clearly demonstrate that the accumulation of glutathione during viral infection activates plant defense, through the expression of *PR-1* genes and SA-mediated, pathways and leads to resistance.

While the positive effects of glutathione on symptom development during viral infections are well established, the role of glutathione on the subcellular level are more difficult to dissect. A tolerant pumpkin cultivar, which did not develop symptoms of ZYMV-disease but contained virions and ZYMV-induced ultrastructural alterations, showed a much stronger increase of glutathione contents in all cell compartments than the susceptible species [53,54]. On the cellular level, the most striking difference can be found in mitochondria where the tolerant species showed a 57% increase of glutathione contents while the susceptible showed a slight decrease (Table 3). These results suggest that the accumulation of glutathione in mitochondria plays an important role in the development of tolerance resulting in a lack of symptom development and decreased virus contents in this cultivar [53]. It was shown during necrotrophic fungal and avirulent bacterial infection that the accumulation of GSSG in mitochondria, a shift towards its oxidized form, and the accumulation of ROS [33,44,58,75] induced mitochondrial dysfunction and activated cell death [33,72,74]. Thus, it is very likely that the same mechanisms can be applied during ZYMV-infection as well. Furthermore, the decrease of glutathione contents in mitochondria led to symptom development in the susceptible species [49], while elevated glutathione levels induced resistance in the tolerant species [48]. In contrast to these results, glutathione contents were decreased in mitochondria of tobacco plants during incompatible TMV-infection 4 dpi while glutathione contents were increased or unchanged in all other cell compartments [58]. These results could be correlated with HR indicating a massive accumulation of ROS resulting in the development of lesions. On top of that, a strong induction of several *NPR* genes and systemic resistance were observed during this interaction [58]. Thus, it is very likely that the accumulation of ROS triggered by insufficient protection of glutathione in mitochondria, activated cell death, which triggered resistance as observed during other plant pathogen interactions [32,33,51].

In this respect, the increase of glutathione contents after ZYMV-infection in the tolerant species was much higher in chloroplasts (163%) and peroxisomes (247%) than in the susceptible species (Table 3) [53]. Tobacco plants susceptible to TMV showed the strongest increase in glutathione contents in chloroplasts and peroxisomes among all cell compartments, 7 dpi with TMV [58]. During viral infections, chloroplasts are especially vulnerable as viruses negatively interfere with photosynthesis, decrease chlorophyll and thylakoid contents, and induce the accumulation of ROS in chloroplasts [56,85,86,87]. Changes in chloroplast ultrastructure such as the decrease in number of chloroplasts, and thylakoid and chlorophyll content, then lead to symptoms such as yellowing and yellow mosaic on the leaves [56,59,87]. Thus, the accumulation of glutathione in chloroplasts (and peroxisomes) during viral infection counteracts ROS accumulation and protects plants from the effects of oxidative damage [53,56,58]. The lack of symptoms of the tolerant ZYMV-infected species, which showed much higher levels of glutathione in chloroplasts and peroxisomes than the susceptible one, supports these claims [53,54]. The importance of glutathione in chloroplasts is also supported by results obtained in OTC-treated plants which showed 135% higher glutathione contents immediately after OTC-treatment, which resulted in a delayed symptom development and decreased virus contents [55]. After ZYMV-infection, chloroplasts in these plants contained 39% higher glutathione levels than untreated ZYMV-infected plants while all other cell compartments showed unchanged glutathione levels Table 3.

Summing up, during compatible plant virus infections, elevated glutathione contents in chloroplasts (and peroxisomes) are essential to counteract ROS accumulation in order to prevent damage to chloroplasts and symptom development. During incompatible plant virus infections the main role of glutathione in mitochondria is to activate plant defense to induce resistance.

## 5. Mitochondria

Mitochondria play essential roles in the protection of plants against biotic stress. Among all cell compartments, mitochondria contain the highest concentrations of glutathione (Figure 2). ROS accumulation in mitochondria is commonly observed during biotic stress and involved in the activation of HR [49,52,76,88,89,90,91]. ROS in mitochondria can lead to mitochondrial dysfunction, which sends retrograde signals to the nuclei, and activates cell death [72,74,91]. ROS produced in mitochondria can also diffuse into the cytosol and the nucleus, where they act as signaling molecules and activate plant defense genes [92,93]. Thus, the severity of ROS accumulation in mitochondria plays an important regulatory role in the response of plant against pathogens. Antioxidants such as glutathione and ascorbate counteract ROS production and regulate defense processes [27,33]. For these reasons, changes in glutathione contents in mitochondria can give valuable information about the regulatory roles of glutathione during biotic stress.

During incompatible TMV-infection in tobacco, the depletion of glutathione in mitochondria was accompanied with the development of necrotic lesions during the HR [58]. A similar situation was found in Arabidopsis plants infected with the necrotrophic fungi *Botrytis cinerea* [52]. Mitochondria were the only organelle which showed decreased levels of glutathione 48 hpi at the infection site. Additionally, glutathione contents remained unchanged in this cell compartment 48 hpi in cells adjacent to the infection site while glutathione contents were increased between 95–358% in all other cell compartments. These results could also be correlated with a strong accumulation of H_2_O_2_ as well as strongly decreased ascorbate contents in mitochondria [52]. Unchanged levels of glutathione and decreased ascorbate contents could also be found in mitochondria in Arabidopsis plants 24 and 48 hpi after the inoculation with the avirulent strain of *Pseudomonas syringae*, which induced necrosis of the leaves and a strong accumulation of ROS at these time points [75]. It has also been shown in *Pseudomonas syringae* infected Arabidopsis plants that an appropriate GSSG status, triggered through oxidative stress, is necessary to induce resistance through the accumulation of SA and the induction of *PR* genes [33]. In tomato plants infected with *Botrytis cinerea*, the strong drop of glutathione contents in mitochondria was accompanied with the accumulation of GSSG and pathogen induced senescence as early as 48 hpi, while ascorbate contents and the activities of APX, MDHAR, DHAR and GR remained at similar levels to the controls [44,94]. These results clearly demonstrate that the depletion of glutathione in mitochondria is a common response of plants during incompatible plant-pathogen interactions and the infection of necrotrophic pathogens. On one side, the accumulation of ROS induced mitochondrial dysfunction and led to cell death during necrotrophic fungal and bacterial infections [43,44,52,94]. On the other side, the ROS-induced depletion of glutathione in mitochondria and a change towards a more oxidative state triggered resistance through SA-mediated pathways during incompatible interactions [33,53,73,75].

Such a clear picture did not evolve in mitochondria during compatible plant pathogen infections when plants failed to develop resistance. Glutathione contents in mitochondria remained unchanged during ZYMV-infection [55], increased during TMV-infection [59], remained unchanged (24 hpi), and slightly decreased (48 hpi) during virulent *Pseudomonas syringae* infection [43,75]. Additionally, glutathione levels mimicked levels of glutathione in other cell compartments during the virulent strain of *Pseudomonas syringae* at the later stages of infection, and ROS accumulation was by far weaker than during the avirulent interaction [75]. It is also interesting that a tolerant pumpkin species that did not develop symptoms of ZYMV-disease, despite containing virus particles and showing ultrastructural alterations, showed strongly elevated glutathione levels in all cell compartments, including mitochondria [53].

These differences in the levels of glutathione in mitochondria between compatible and incompatible plant pathogen interactions demonstrate that glutathione plays an important regulatory role in mitochondrial defense against pathogens. During incompatible plant-pathogen interaction, the depletion of glutathione in mitochondria and a change towards GSSG led to the accumulation of ROS [33,43,44,58,75,94], which resulted to mitochondrial dysfunction, activated cell death, initiated plant defense, and triggered resistance [72,74]. A similar pattern occurred during the infection of Arabidopsis and tomato plants during infection of the necrotrophic pathogen *Botrytis cinerea*, where the breakdown of the antioxidative system led to the accumulation of ROS and cell death but did not trigger resistance [44,52]. During compatible plant interactions, which result in symptoms but not in cell death, glutathione levels in mitochondria either increase, or mimic, the situation in all other cell compartments [59,75]. The primary role of glutathione in mitochondria during such interactions seems to be to keep ROS under control in order to avoid damage to cellular components and cell death.

## 6. Chloroplasts and Peroxisomes

Chloroplasts and peroxisomes are important organelles for ROS generation and defense signaling. ROS produced in the chloroplasts, for example, are involved in the induction of HR [49,76,95]. Peroxisomes are crucial in detoxifying ROS in plants as they accumulate catalase, which detoxifies H_2_O_2_ [73]. In this context, the importance of peroxisomes for plant defense is highlighted by catalase deficient Arabidopsis and tobacco mutants. These mutants contain enhanced photorespiratory H_2_O_2_ levels which trigger the induction of pathogen related defense genes and cell death [21,33,73,78,79]. Under these circumstances, the activation of plant defense during *Pseudomonas syringae* infection was correlated with the accumulation of GSSG and the activation of SA signaling, which led to reduced bacterial growth [33]. Blocking glutathione oxidation and accumulation induced by H_2_O_2_ strongly reduced plant defense, lesion formations, and led to bacterial growth similar to the control [33]. These results clearly indicate that in situations of catalase deficiency in peroxisomes, GSSG and H_2_O_2_ accumulate and activate plant defense through SA-mediated pathways.

The importance of glutathione in chloroplasts and peroxisomes for plant defense are further supported by the massive increase of glutathione of up to 133% and 452%, respectively, during the early stages of *Pseudomonas syringae* infection in Arabidopsis. The breakdown of the antioxidative system in these two cell compartments at the later stages of infection correlated with the accumulation of ROS and progress of advanced disease symptoms [75]. In tobacco plants infected with *Botrytis cinerea*, a strong increase of glutathione was detected in both chloroplasts (up to 358%) and peroxisomes (up to 227%) 12 hpi and 48 hpi after the inoculation at the infection site and in cells adjacent of the infection site, respectively [52]. Within the cell, the chloroplast was the first cell compartment (together with mitochondria) that showed an accumulation of ROS [52]. ROS accumulation in chloroplasts is also a common feature during viral infections [56,85]. As glutathione detoxifies ROS, it is not surprising that tobacco plants susceptible to TMV showed the strongest increase of glutathione contents in chloroplasts and peroxisomes 7 dpi [58]. Peroxisomes also showed the strongest increase of glutathione contents in a tolerant pumpkin species infected with ZYMV [53]. While elevated glutathione contents in chloroplasts and peroxisomes are important to control ROS and to contain the development of symptoms at the early stages of infection, the breakdown of the antioxidative system in these two organelles can be correlated with the development of symptoms such as necrosis and senescence in *Botrytis cinerea* infected Arabidopsis and tobacco plants [44,52]. In both interactions, glutathione and ascorbate contents strongly decreased at the final stages of infection, and the ratio of both shifted towards their oxidized forms [44,52]. Additionally, the activities of APX, DHAR, MDHAR, and APX decreased, or remained at control values, in both organelles at the final stages of infection [44]. The results of these studies clearly demonstrate that high levels of glutathione in chloroplasts and peroxisomes suppress ROS accumulation during various different plant pathogen interactions and counteract symptom development at the beginning of infections. When glutathione fails to keep ROS in these two cell compartments under control, systemic symptoms and cell death occur [44,52,56,75,76].

Besides the accumulation of glutathione in chloroplasts and peroxisomes during pathogen attack, its redox state is also of great importance for the development of resistance. It has been demonstrated that a plastidial GR enhances resistance in wheat against *Blumeria graminis* [51]. It was concluded by the authors that GR increased the ratio of glutathione towards its reduced form. The change of the cytosolic redox state reduced NPR1 to a monomer, which when imported into the nucleus subsequently led to the activation of SA-mediated *PR* genes [51]. A similar result was found in transgenic tobacco plants inoculated with *Pseudomonas syringae*, where the introduction of chloroplastic targeted GSH1 increased glutathione contents and its ratio towards GSH. This change activated SA-related defense genes and genes involved in ethylene synthesis, which decreased symptom development [32].

Summing up, these results demonstrate that if glutathione fails to keep ROS in chloroplasts and peroxisomes under control systemic symptoms and cell death occur. Changes in the redox state of glutathione induced by ROS in chloroplasts and peroxisomes initiate the activation of defense genes through SA-mediated pathways.

## 7. Apoplast

The apoplast of plant cells is the first line of defense against pathogens. Thus, the accumulation of ROS in the apoplast is a commonly observed reaction, which is aimed to either kill the pathogen directly, or to induce local cell death of infected tissue in order to stop the spread of the dangerous invader [4,71]. The activity and levels of antioxidants are generally low in the apoplast. This allows ROS to accumulate and oxidize rapidly, which is an important condition for ROS signaling. Glutathione and ascorbate have not been detected in the apoplast during TMV- and ZYMV-infection in tobacco and pumpkin plants, respectively [54,58,59]. The same situation was found in Arabidopsis plants infected with the bacterial pathogen *Pseudomonas syringae* and the necrotrophic fungal pathogen *Botrytis cinerea* [52,75]. These results are surprising because in both interactions a strong accumulation of ROS was observed. H_2_O_2_ accumulation was especially pronounced in the apoplast during the early stages of *Botrytis cinerea* infection [52]. Very low contents of glutathione and ascorbate were detected in the apoplast of barley and oat plants during the infection of the biotrophic fungi *Blumeria graminis* [45,46]. While ascorbate contents decreased in both the resistant and the susceptible species, glutathione contents increased and its ratio shifted towards GSSG in the resistant species, but it decreased in the susceptible one. The activities of GR, APX, MDHAR, DHAR were similar or even more pronounced in the susceptible species when compared to the resistant one. As antioxidative defense mechanisms were not able to prevent H_2_O_2_-induced cell death, the increase of glutathione, and its shift towards GSSG in the apoplast during *Blumeria graminis* infection, is to activate plant defense signaling rather than to detoxify H_2_O_2_ [45,46]. Similar results were obtained in tobacco plants inoculated with powdery mildew, where the artificial elevation of glutathione through injection of GSH into the apoplast enhanced basal resistance against *Euoidium longipes* [34]. A signaling role for apoplastic glutathione during *Blumeria graminis* infection is supported by another study with the same plant-pathogen constellation. This study came to the conclusion that high ratio of GSH/GSSG was required for proper plant defense and that GR mediated the activation of the *NPR1* gene [51]. On top of that several other studies concluded that changes in the redox state of glutathione trigger the activation of plant defense leading to resistance [32,33].

The reasons for low apoplastic glutathione contents during biotic stress can be found in a high degradative activity of GGT1 and GGT2, which are responsible for glutathione degradation. They are both located in the cell wall and the plasma membrane, respectively. In *ggt1* knock out mutants, where glutathione degradation in the apoplast is not performed properly, apoplastic glutathione levels were found to be similar to glutathione contents in plastids [64]. These changes of apoplastic glutathione contents were associated with modifications of the proteome that were similar to those found during abiotic and biotic stress conditions. These results are another indicator that glutathione contents and the redox state in the apoplast are involved in sensing and signaling environmental stress, thus have a key role in the adaption of plants to biotic stress.

## 8. Cytosol

The cytosol can be considered to be the switch board of the cell during biotic stress. During biotic stress situations, ROS diffuse into the cytosol and signal agents are sent from the different cell compartments through the cytosol into the nuclei. Transcripts from the nuclei get transported into the cytosol, distributed to other cell compartments, and translated into proteins. Various different agents (effectors, hormones, salicylic acid, antioxidants, etc.) accumulate in the cytosol and are distributed to the different cell organelles [8,26,27,28,29,32,33,36,37,38,96]. In this context, the cytosol plays many essential roles for glutathione metabolism during plant defense. The most important one is to synthesize glutathione which is then transported to the different cell compartments to assuage the higher demand for glutathione during biotic stress. While the first step of glutathione synthesis, which links glutamate with glycine, takes place exclusively in the chloroplasts, the second step, which adds cysteine, takes place in chloroplasts and the cytosol [61]. Interestingly, it has been shown that restricting the last step of glutathione synthesis to the cytosol is sufficient for proper plant development [97] and that chloroplasts import glutathione from the cytosol through specific transporters [65]. Increased glutathione synthesis, accumulation of glutathione in the cytosol, and transportation to the different cell compartments is especially crucial at the early phase of pathogen attack when most cell compartments accumulate glutathione as demonstrated during bacterial, fungal, and viral infections [52,58,59,75]. On top of that, boosting glutathione synthesis in the cytosol, by increasing the availability of the rate limiting precursor cysteine, induced different levels of resistance during various viral infections. The artificial increase of glutathione by OTC and sulfur treatment inhibited symptom development and decreased virus contents in ZYMV-infected pumpkin plants [54,55] and TMV-infected tobacco plants [58,59], respectively. OTC pretreatment also induced partial protection against PPV in pea plants [56]. Elevated glutathione contents could be correlated in tobacco plants with the activation of defense related genes in nuclei and accumulation of related proteins [58,59]. In all of these studies, glutathione levels in the cytosol closely mimicked the situation of the majority of the other cell compartments, indicating that most cell compartments use the cytosol as a source for glutathione at the early stages of infection. As glutathione does not accumulate inside the cytosol at the later stages of infection when glutathione gets depleted in most other organelles, it appears that glutathione gets transported quite efficiently through the cytosol to the plasma membranes and vacuoles, which contain peptidases and facilitate the degradation of glutathione [62,63,64].

Glutathione in the cytosol plays important signaling roles during biotic stress. It has been demonstrated that the accumulation of glutathione in the cytosol of tobacco plants can be linked to strongly increased transcripts of agents (NPR1, PR1, MAPKK) involved in plant defense [98]. Similar results were obtained in tobacco plants infected with TMV where the artificial elevation of glutathione in the cytosol through the external application of GSH or OTC induced resistance through the overexpression of *PR* genes in nuclei and salicylic acid mediated pathways [35]. Treatment of Arabidopsis seedlings with glutathione activated several genes involved in plant defense signaling and in jasmonic acid biosynthesis [38]. Another study showed that GSH treatment activated several biotic stress- and ethylene related genes in nuclei as well as the accumulation of stress and defense signaling proteins [99]. Furthermore, the application of glutathione up-regulated the biosynthesis of abscisic acid, auxin, and JA [37]. On the other hand, it has also been demonstrated that Arabidopsis mutants deficient in glutathione showed enhanced susceptibility to pathogens indicating the importance of sufficient supply of glutathione for successful plant defense [100,101,102]. Additionally, through the use of several GSH-deficient Arabidopsis mutants it was demonstrated that an appropriate oxidized glutathione status in the cytosol during *Pseudomonas syringae* infection triggered plant resistance through the accumulation of SA and the induction of *PR* genes in the nucleus [33]. An important role has also been assigned for H_2_O_2_ during these studies. It was concluded by the authors that increased intracellular H_2_O_2_ activated SA signaling [33]. In contrast, the overexpression of a chloroplastic GR in wheat, which increased the ratio of GSH/GSSG in the cytosol, resulted in enhanced resistance against *Blumeria graminis* through the induction of pathogen related *PR-1* and *PR-5* genes [51]. The authors concluded that the enhanced capability of converting GSSG to GSH resulted in increased resistance by protecting cells against oxidative stress. The reduction of NPR1 in the cytosol activated the expression of SA-mediated *PR* genes, which subsequently regulated systemic acquired resistance [51,103].

Summing up, the above studies indicate that the main role of the cytosol during biotic stress is to provide enough glutathione to counteract ROS production throughout the cell and to distribute it equally among the cell compartments. Pathogen-induced changes in the redox state of glutathione in the cytosol can then trigger various pathways of plant defense through the activation of defense genes.

## 9. Nuclei

As outlined in the previous chapter, the interaction between the nuclei and the cytosol is crucial for successful plant defense as signals processed in the cytosol lead to the activation of defense genes in nuclei. The transcripts are then sent from the nuclei into the cytosol and other organelles where they lead to metabolic changes that can trigger plant defense and resistance [8,25,26,27,28,31,32,35,36,37,88]. ROS and glutathione play important roles by mediating the above described processes. During biotic stress situations, ROS and GSSG generated in the different organelles diffuse, or get transported, respectively, into the cytosol where they can change the redox state of glutathione and modify proteins that are then sent to the nucleus where they activate the expression of plant defense genes [27,33,50,98]. While ROS can also diffuse into nuclei and directly activate the expression of defense genes such effects have not been demonstrated for glutathione yet [104]. It seems that the mediating roles of glutathione during biotic stress in plants are mainly restricted to the cytosol and other organelles.

In most plant pathogen interactions, glutathione content in nuclei mimicked the situation in the cytosol indicating that glutathione simply diffuses into the nuclei after the production in the cytosol [52,53,59,105]. Differences in glutathione contents between the cytosol and nuclei were consistently found at the later stages during incompatible plant pathogen interaction when HR resulted in the accumulation of ROS, necrosis, and development of resistance [58,75]. In resistant tobacco plants infected with TMV, the nucleus showed a slight increase of glutathione while the cytosol showed unchanged levels 48 hpi [58]. During the infection of Arabidopsis plants, with an avirulent strain of *Pseudomonas syringae*, the nucleus showed a strong increase in glutathione contents while the cytosol contained decreased levels 48 hpi (Table 2). Based on these results, it is tempting to assume that glutathione is involved in redox regulation of nuclear gene expression that leads to resistance. Nevertheless, an accumulation of glutathione in nuclei was also found in Arabidopsis plants infected with a virulent strain of *Pseudomonas syringa* [75]. In the final stages of this infection, when ROS started to accumulate in the leaves, glutathione strongly increased in the nuclei and remained unchanged in the cytosol (Table 2). From these results and the observation that glutathione levels in nuclei simply mimic the situation in the cytosol during most plant pathogen interactions [52,53,59,105], it seems that glutathione contents do not directly impact gene expression inside nuclei. Nevertheless, it has been demonstrated that glutathione levels in nuclei indirectly contribute to plant defense by providing reducing moiety for antioxidant enzymes which are able to modify transcription factors and proteins involved in plant defense [106,107,108,109,110,111]. On top of that, it has also been demonstrated that glutathione is essential to maintain the redox balance in nuclei. Alterations of the redox state restricts nuclear functions and impairs progression through the cell cycle [112,113,114,115]. Even though such effects still have to be clearly demonstrated during biotic stress, it is very likely that glutathione also acts as an important redox buffer in nuclei during biotic stress and indirectly regulates gene expression by controlling ROS and keeping the nucleus in a reduced state.

## 10. Concluding Remarks

**Figure 3:**
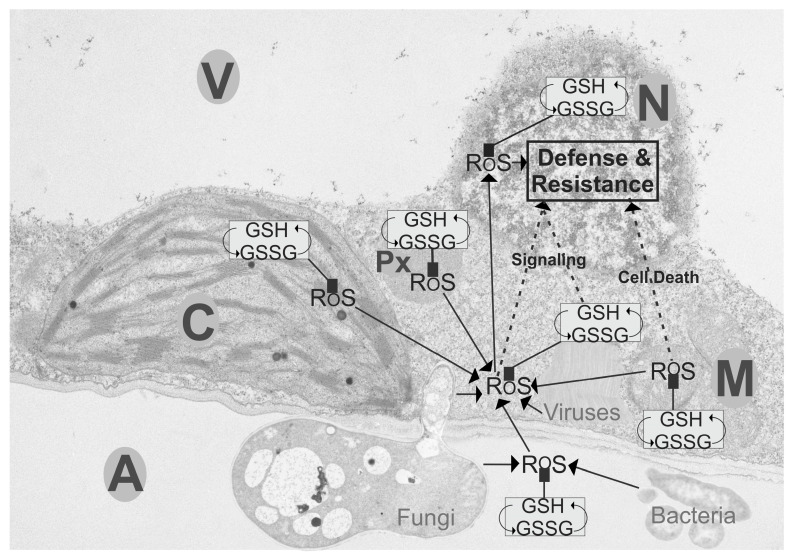
Compartment-specific roles of glutathione and reactive oxygen species (ROS) during plant defense. Transmission electron micrograph of a leaf cell with overlay demonstrating the roles of glutathione (GSH, GSSG) and ROS in the activation of plant defense during biotic stress. The first line of defense in plants is the apoplast, where during biotrophic fungal infection, the accumulation of ROS and GSSG activates plant defense and leads to resistance. During pathogen attack, the depletion of glutathione in mitochondria and a change towards GSSG leads to the accumulation of ROS, which causes mitochondrial dysfunction, activates cell death, and triggers resistance. The accumulation of glutathione in chloroplasts and peroxisomes at the early stages of plant pathogen interactions is related to increased tolerance and the development of resistance. The collapse of the antioxidative system in both compartments at the later stages of infections enforces cell death through retrograde signaling. During biotic stress situations ROS and GSSG generated in the different organelles diffuse, or get transported, respectively, into the cytosol where they can change the redox state of glutathione and modify proteins. Proteins are then sent to the nucleus, where they activate the expression of defense gene through pathways that involve SA, JA, auxins, and abscisic acid. While ROS can also diffuse into nuclei and directly activate the expression of defense genes, such effects have not been demonstrated for glutathione yet. Within nuclei glutathione mediates gene expression through detoxifying ROS and acting as a redox buffer for antioxidant enzymes, which can then lead to modifications of transcription factors and activation of defense gene expression. A, apoplast, C, chloroplasts, M, mitochondria, N, nuclei, Px, peroxisomes, V, vacuoles.

Plant defense against biotic stress relies on the communication of all cell compartments and involves sensing, signaling, and activating plant defense. Subcellular glutathione and ROS contents play important roles in these processes as outlined in this review (Figure 3). While glutathione in the apoplast does not play an important role during virus, bacterial, and necrotrophic fungal infection, its redox state and the accumulation of ROS in the apoplast during the infection of biotrophic fungi initiates the activation of plant defense leading to resistance. During incompatible plant-pathogen interaction, the depletion of glutathione in mitochondria and a change towards GSSG leads to the accumulation of ROS, which then leads to mitochondrial dysfunction, activates cell death, and triggers resistance. During compatible plant interactions the primary role of glutathione in mitochondria is to keep ROS under control in order to avoid damage to mitochondrial components and inhibit cell death. The accumulation of glutathione in chloroplasts and peroxisomes at the early stages of plant pathogen interactions was related to increased tolerance and the development of resistance. The collapse of the antioxidative system in these two cell compartments at the later stages enforced cell death through retrograde signaling. ROS and glutathione in the cytosol and nuclei play important roles by mediating plant defense. During biotic stress situations, ROS and GSSG generated in the different organelles diffuse, or get transported, respectively, into the cytosol, where they can change the redox state of glutathione and modify proteins that are then sent to the nucleus. They then activate the expression of defense genes through pathways that involve SA, JA, auxins, and abscisic acid. While ROS can also diffuse into nuclei and directly activate the expression of defense genes, such effects have not been demonstrated for glutathione yet. It seems that glutathione within nuclei indirectly mediates gene expression by detoxifying ROS and acting as a redox buffer for antioxidant enzymes, which can then lead to modifications of transcription factors and activation of defense gene expression.

## Figures and Tables

**Table 1:** Changes in percentage of subcellular glutathione contents in Arabidopsis infected with *Botrytis cinerea*. Glutathione contents were determined at different hours, post inoculation (hpi), in cells at the infection site (tissue underneath the drop containing spores), adjacent to the infection site, and compared to the control [52]. Significant differences were calculated using the Mann-Whitney U-test; ** and ***, respectively, indicate significance at the 0.01 and 0.001 levels of confidence; ns = not significantly different.

	*Botrytis cinerea*			
	Infection site		Adjacent to Infection Site	
	12 hpi	48 hpi	12 hpi	48 hpi
Mitochondria	ns	−39 ***	ns	ns
Chloroplasts	141 ***	92 ***	ns	358 ***
Nuclei	79 ***	ns	ns	132 ***
Peroxisomes	122 **	ns	93 ***	227 ***
Cytosol	132 **	ns	ns	95 ***

**Table 2:** Changes in percentage of subcellular glutathione contents in Arabidopsis infected with *Pseudomonas syringae*. Glutathione contents were determined at different hours post inoculation (hpi) in plants infected with a virulent and avirulent strain of *Pseudomonas syringae* pv. *tomato* DC3000 and compared to the control [75]. Significant differences were calculated with the Kruskal-Wallis test followed by post-hoc comparison according to Conover at *p* < 0.05. * indicates significance at the 0.05 level of confidence; ns = not significantly different.

	*Pseudomonas syringae*					
	Virulent			Avirulent		
	12 hpi	24 hpi	48 hpi	12 hpi	24 hpi	48 hpi
Mitochondria	44 *	ns	−29 *	29 *	ns	ns
Chloroplasts	73 *	133 *	−48 *	25 *	ns	−40 *
Nuclei	ns	158 *	70 *	53 *	70 *	38 *
Peroxisomes	ns	452 *	−48 *	139 *	258 *	−39 *
Cytosol	ns	307 *	ns	143 *	160 *	−41 *

**Table 3:** Changes in percentage of subcellular glutathione contents during Zucchini Yellow Mosaic Virus (ZYMV) and Tobacco Mosaic Virus (TMV) infection. Glutathione contents were determined at different days post inoculation (dpi) in OTC-treated, susceptible, and tolerant ZYMV-infected pumpkin and susceptible and resistant TMV-infected tobacco plants and compared to the control [55,58,59]. Significant differences were calculated using the Mann-Whitney U-test; *, ** and ***, respectively, indicate significance at the 0.05, 0.01 and 0.001 levels of confidence; ns = not significantly different. OTC = L-2-oxothiazolidine-4-carboxylic acid.

	ZYMV			TMV			
	Susceptible (14 dpi)		Tolerant (14 dpi)	Susceptible		Resistant	
		OTC		7 dpi	14 dpi	1 dpi	4 dpi
Mitochondria	ns	ns	57 **	125 ***	35.8 **	ns	−17 *
Chloroplasts	50 *	39 *	163 ***	246 ***	−23.4 *	ns	39 *
Nuclei	115 *	ns	202 ***	203 ***	ns	26 *	28 *
Peroxisomes	81 *	ns	247 ***	265 ***	88 ***	ns	ns
Cytosol	195 *	ns	163 ***	195 ***	ns	ns	ns
